# Longitudinal Nasopharyngeal Carriage and Antibiotic Resistance of Respiratory Bacteria in Indigenous Australian and Alaska Native Children with Bronchiectasis

**DOI:** 10.1371/journal.pone.0070478

**Published:** 2013-08-05

**Authors:** Kim M. Hare, Rosalyn J. Singleton, Keith Grimwood, Patricia C. Valery, Allen C. Cheng, Peter S. Morris, Amanda J. Leach, Heidi C. Smith-Vaughan, Mark Chatfield, Greg Redding, Alisa L. Reasonover, Gabrielle B. McCallum, Lori Chikoyak, Malcolm I. McDonald, Ngiare Brown, Paul J. Torzillo, Anne B. Chang

**Affiliations:** 1 Menzies School of Health Research, Charles Darwin University, Darwin, Northern Territory, Australia; 2 Alaska Native Tribal Health Consortium, Anchorage, Alaska, United States of America; 3 Centers for Disease Control and Prevention, Arctic Investigations Program, National Center for Emerging and Zoonotic Infectious Diseases, Anchorage, Alaska, United States of America; 4 Queensland Children's Medical Research Institute, The University of Queensland, Brisbane, Australia; 5 Department of Infectious Diseases, Royal Children's Hospital, Brisbane, Queensland, Australia; 6 Department of Epidemiology and Preventive Medicine, Monash University, Melbourne, Victoria, Australia; 7 Infectious Diseases Unit, Alfred Hospital, Melbourne, Victoria, Australia; 8 Royal Darwin Hospital, Darwin, Northern Territory, Australia; 9 School of Medicine, University of Washington, Seattle, Washington, United States of America; 10 Pulmonary Division, Children's Hospital and Regional Medical Center, Seattle, Washington, United States of America; 11 Yukon Kuskokwim Health Corporation, Bethel, Alaska, United States of America; 12 School of Medicine and Dentistry, James Cook University, Cairns, Queensland, Australia; 13 University of Wollongong, Wollongong, New South Wales, Australia; 14 Australian Indigenous Doctors' Association, Parkes, Australian Capital Territory, Australia; 15 Nganampa Health Council, Alice Springs, Northern Territory, Australia; 16 Royal Prince Alfred Hospital, Sydney, New South Wales, Australia; 17 Department of Medicine, University of Sydney, Sydney, New South Wales, Australia; 18 Department of Respiratory Medicine, Queensland Children's Medical Research Institute, Royal Children's Hospital, Brisbane, Queensland, Australia; Centers for Disease Control & Prevention, United States of America

## Abstract

**Background:**

Indigenous children in Australia and Alaska have very high rates of chronic suppurative lung disease (CSLD)/bronchiectasis. Antibiotics, including frequent or long-term azithromycin in Australia and short-term beta-lactam therapy in both countries, are often prescribed to treat these patients. In the Bronchiectasis Observational Study we examined over several years the nasopharyngeal carriage and antibiotic resistance of respiratory bacteria in these two PCV7-vaccinated populations.

**Methods:**

Indigenous children aged 0.5–8.9 years with CSLD/bronchiectasis from remote Australia (n = 79) and Alaska (n = 41) were enrolled in a prospective cohort study during 2004–8. At scheduled study visits until 2010 antibiotic use in the preceding 2-weeks was recorded and nasopharyngeal swabs collected for culture and antimicrobial susceptibility testing. Analysis of respiratory bacterial carriage and antibiotic resistance was by baseline and final swabs, and total swabs by year.

**Results:**

*Streptococcus pneumoniae* carriage changed little over time. In contrast, carriage of *Haemophilus influenzae* declined and *Staphylococcus aureus* increased (from 0% in 2005–6 to 23% in 2010 in Alaskan children); these changes were associated with increasing age. *Moraxella catarrhalis* carriage declined significantly in Australian, but not Alaskan, children (from 64% in 2004–6 to 11% in 2010). While beta-lactam antibiotic use was similar in the two cohorts, Australian children received more azithromycin. Macrolide resistance was significantly higher in Australian compared to Alaskan children, while *H. influenzae* beta-lactam resistance was higher in Alaskan children. Azithromycin use coincided significantly with reduced carriage of *S. pneumoniae, H. influenzae* and *M. catarrhalis*, but increased carriage of *S. aureus* and macrolide-resistant strains of *S. pneumoniae* and *S. aureus* (proportion of carriers and all swabs), in a ‘cumulative dose-response’ relationship.

**Conclusions:**

Over time, similar (possibly age-related) changes in nasopharyngeal bacterial carriage were observed in Australian and Alaskan children with CSLD/bronchiectasis. However, there were also significant frequency-dependent differences in carriage and antibiotic resistance that coincided with azithromycin use.

## Introduction

Indigenous children in Australia, Alaska and New Zealand have amongst the world's highest recorded rates of chronic suppurative lung disease (CSLD), including bronchiectasis unrelated to cystic fibrosis (CF) [1]. Antibiotics play a key role in managing persistent respiratory symptoms and acute exacerbations associated with bronchiectasis [2]. However, few longitudinal data exist on the impact of antibiotics upon the airway bacteriology of these patients. This is an important knowledge gap as Indigenous children have high rates of nasopharyngeal bacterial carriage [3] and microaspiration of upper airway bacteria may contribute to both the pathogenesis and on-going morbidity of bronchiectasis [4].

Based upon the clinical impression that patients receiving azithromycin have fewer acute respiratory exacerbations, there is anecdotal evidence that many clinicians in Australia routinely prescribe azithromycin (frequently long-term) for children with bronchiectasis, while in Alaska such practice is uncommon. In contrast, both sites utilize short-term intermittent therapy with beta-lactam antibiotics (e.g. amoxicillin or amoxicillin-clavulanate) for acute pulmonary exacerbations associated with bronchiectasis. The Bronchiectasis Observational Study was established in Australia and Alaska to study prospectively the clinical course of CSLD/bronchiectasis in Indigenous children [1]. Using the opportunity thus provided, we examined the nasopharyngeal carriage of respiratory bacteria and their antibiotic resistance patterns in children. We hypothesised that the two populations would differ in their nasopharyngeal carriage of potential respiratory bacterial pathogens and antibiotic resistance in these organisms as a result of differences in prescribing practices between the two settings.

## Methods

### Subjects

Children 0.5–8.9 years of age with a diagnosis of non-CF bronchiectasis (confirmed on high-resolution computerized tomography) or chronic (>3-months) daily wet (or productive) cough were enrolled in a prospective cohort study during 2004–8 [1]. Children were recruited opportunistically while attending an outpatient clinic or in hospital undergoing bronchoscopy for suspected CSLD/bronchiectasis or for respiratory exacerbations. Subsequently, enrolled children were examined and nasopharyngeal swabs collected by research staff at scheduled study visits until 2010. These visits were scheduled quarterly in Australia (to ensure at least one annual review in highly mobile enrolled children) and annually in Alaska. Such visits were planned independently of the children's health status. Only visits where swabs were collected were included in this analysis. Children's vaccination status at baseline was recorded from immunisation registers. Antibiotic use in the 2-weeks preceding swab collection (to capture recent antibiotic events and allow comparison with other studies in this population [5] and studies using parent recall) was recorded from clinic notes.

### Ethics statement

The study was approved by the Human Research Ethics Committee of the Northern Territory Department of Health and Menzies School of Health Research in Australia (HREC 04/46) and the Alaska Area Institutional Review Board (which acts under the Indian Health Service Institutional Review Board FWA number 0008894). At enrolment written informed consent was obtained from the carer of each child and when appropriate assent was also provided by the older children in the cohort. The process of obtaining consent, and the information sheets and consent forms used (stored securely at each research institute), were approved by the respective ethics bodies.

### Specimen collection and laboratory testing

Nasopharyngeal swabs collected at study visits were transported and processed according to published methods [5], [6], unless otherwise noted. Swabs stored in skim-milk tryptone glucose glycerol broth at −80°C were thawed and 10 µL aliquots plated on selective media and incubated overnight at 37°C and 5% CO_2_. In Australia, four colonies each (including any with differing morphologies) of presumptive *Streptococcus pneumoniae* and *Haemophilus influenzae*, two of *Moraxella catarrhalis* and one of *Staphylococcus aureus* were isolated and identified using standard methods [5]. In Alaska, multiple colonies were picked when differing morphologies were observed. When multiple isolates underwent antibiotic susceptibility testing, the child was reported as carrying a resistant strain if one or more of the isolates proved resistant. *S. pneumoniae* serotypes were determined by the Quellung reaction using antisera from Statens Serum Institute (Denmark).

Antimicrobial sensitivities for *S. pneumoniae*, *H. influenzae* and *S. aureus* isolates were determined by disk diffusion (Australia [7]; Alaska [8]). Minimum inhibitory concentrations (MICs) were determined for resistant *S. pneumoniae* and *H. influenzae* isolates using Etest strips (AB bioMérieux, France) in Australia. In Alaska, erythromycin and penicillin MICs for *S. pneumoniae* were determined by microbroth dilution and ampicillin MICs for *H. influenzae* were determined using Etest strips. MIC breakpoints from the European Committee on Antimicrobial Susceptibility Testing (EUCAST, http://www.eucast.org) were used to define resistance for *S. pneumoniae* (penicillin resistance MIC>2 mg/L, intermediate resistance MIC>0.06–2 mg/L; macrolide [azithromycin or erythromycin] resistance MIC>0.5 mg/L) and *H. influenzae* (ampicillin resistance MIC>1 mg/L; macrolide resistance MIC>4 mg/L, intermediate resistance MIC>0.12–4 mg/L). A nitrocephin-based test detected beta-lactamase activity in *H. influenzae* and *M. catarrhalis* isolates.

### Data analysis

Carriage data are reported as the proportion of children at baseline (first swab) and as they left the study (last swab), and also presented graphically as the proportion of total swabs collected per study year. As storage of 29 swabs from Central Australia during 2004–6 was at −20°C, which is suboptimal for recovery of *H. influenzae* [9], these samples were excluded from analyses involving this pathogen and the first subsequent swab for each child was included for *H. influenzae* baseline data. Also, as only 20 swabs were collected from both cohorts in 2004–5, their data were combined with those from 2006.

Antibiotic resistance data are reported as the proportion of carriers as children entered and left the study, proportion of total isolates for each bacterial species, and graphically as the proportion of all positive swabs by study year. Multiple strains detected in the same swab (differing serotype or antibiotype for *S. pneumoniae* and *H. influenzae*, differing beta-lactamase status for *M. catarrhalis*) were counted as additional isolates where results for total isolates are presented.

Confidence intervals (CIs) were calculated using the exact binomial method for first and last swabs from each child and where numbers were small (<5), and otherwise adjusted for repeated sampling using the clustered sandwich estimator for estimating the variance-covariance matrix, implemented as vce(cluster *id*) in Stata. We used Stata's logistic command for bivariate and multivariate analyses, and nptrend to test for trend across ordered groups. Statistical tests were performed using Stata 12 (College Station, Texas).

To investigate the cumulative effect of repeated and sustained long-term azithromycin exposure on bacterial carriage and resistance, Australian children were divided into three groups based on frequency of azithromycin use during the study period: Azi-None  =  no azithromycin in the 2-weeks preceding swab collection at any of the study visits; Azi-Infrequent  =  azithromycin preceding 1–50% of study visits; Azi-Frequent  =  azithromycin preceding >50% of study visits. Tables and figures are presented as Supporting Information.

## Results

### Subjects

A total of 120 children were enrolled and 597 swabs collected (Table 1). At enrolment, 24% of Alaskan and 30% of Australian children had respiratory exacerbations. At subsequent visits the numbers were 8% and 17% respectively. The mean number of swabs collected per child per year enrolled (person-years of follow-up) was 2.5 in Australia and 1.3 in Alaska. Most (88%) children had received ≥3 doses of the 7-valent pneumococcal conjugate vaccine (PCV7). In addition, two Australian children received ≥1 dose of the pneumococcal *Haemophilus influenzae* protein D conjugate vaccine introduced in 2009. No Alaskan children received PCV13, introduced in 2010. Australian children were more likely to receive azithromycin in the 2-weeks preceding swab collection, while beta-lactam antibiotic use was similar in the two cohorts (Table 1). There were no clear temporal trends in antibiotic use; peaks were observed in beta-lactam antibiotic and azithromycin use in 2007 and 2008 (preceding 18% and 55% of swabs collected respectively) in Australian children, and in beta-lactam antibiotic use in Alaskan children in 2008 (preceding 25% of swabs collected).

**Table 1:** Enrolment, swab collection, vaccination and antibiotic use in Australian Indigenous and Alaska Native children.

	Australia	Alaska	Total
Children enrolled	79	41	120
Median age at enrolment in years (range)	2.7 (0.8–8.9)	2.8 (0.5–7.9)	
Person-years of follow-up	179	121	300
Median time in study in years (range)	2.2 (0–5.8)	3.4 (0–4.8)	
Number of children who had received ≥3 doses of 7-valent PCV* at enrolment	71 (90%)	35 (85%)	106 (88%)
Swabs collected	443	154	597
Median number of swabs collected (range)	5 (1–15)	4 (1–11)	
Number of swabs where children received antibiotics in the 2-weeks preceding collection:			
Azithromycin	192 (43%)	1 (<1%)	193 (32%)
Beta-lactam antibiotics	44 (10%)	14 (9%)	58 (10%)

* Pneumococcal conjugate vaccine.

### Nasopharyngeal carriage over time

**Figure 1:**
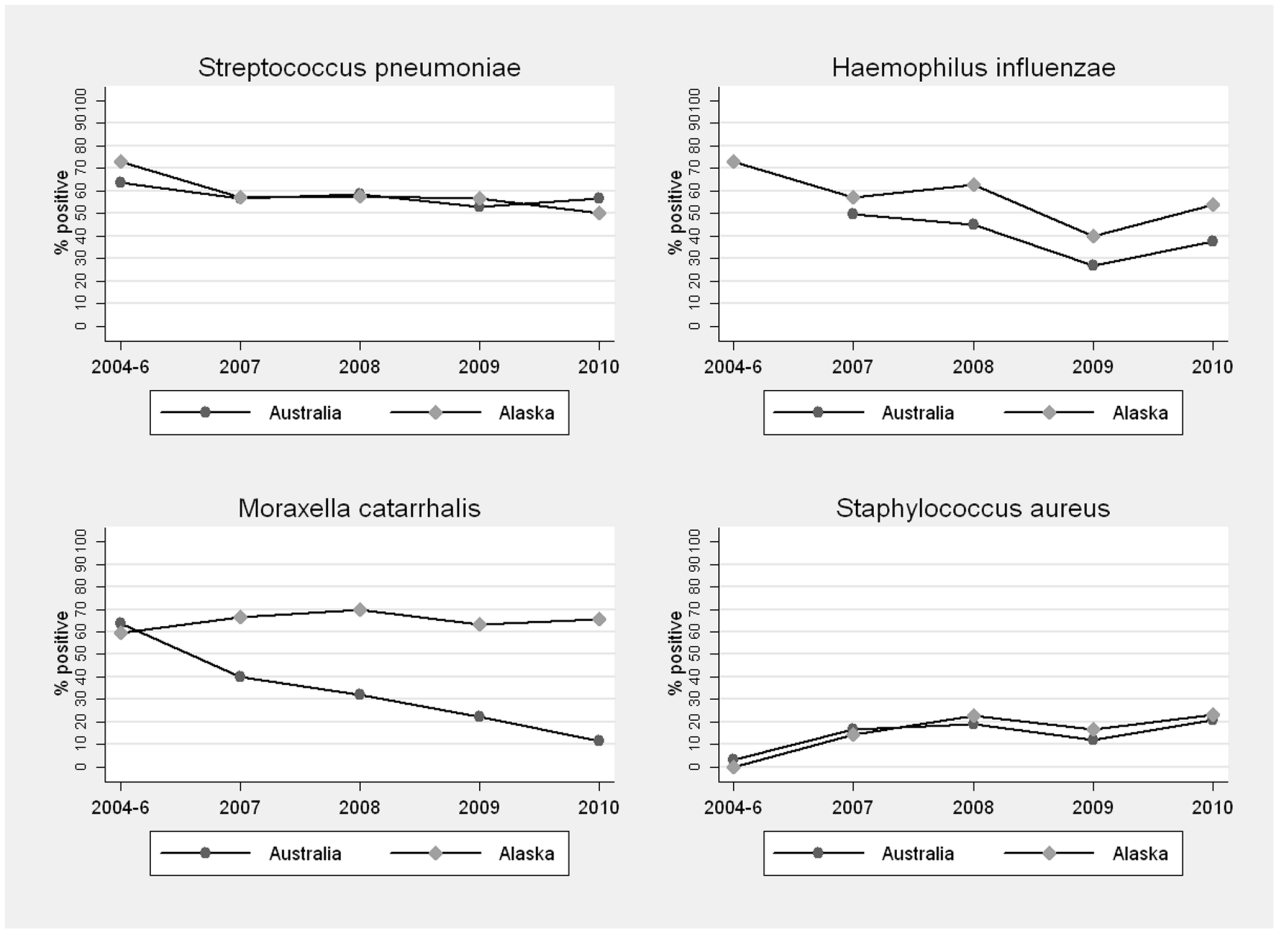
Pathogen carriage (proportion of swabs) by study year in Australian and Alaskan children.

Nasopharyngeal bacterial carriage as children entered and left the study is shown in Table 2. Australian children were at significantly lower risk than Alaskan children of *M. catarrhalis* carriage at the study's end, and this finding persisted after adjusting for carriage at baseline. When all data were analysed as a proportion of swabs by year (Figure 1), *S. pneumoniae* carriage remained relatively stable, *H. influenzae* carriage declined in both Australian (nptrend P = 0.007) and Alaskan (P = 0.033) children, *M. catarrhalis* carriage in Australian children showed a marked decline from 64% (95% CI 47–81) in 2004–6 to 11% (95% CI 2–21) in 2010 (P<0.001), and *S. aureus* carriage in Alaskan children increased from 0% (95% CI 0–9) in 2005–6 to 23% (95% CI 6–40) in 2010 (P = 0.010).

**Table 2:** Nasopharyngeal carriage of respiratory bacteria from Australian and Alaskan children at baseline and end of study.

	First swab for each child		Last swab for each child^1^		
	Australia	Alaska	Australia	Alaska	OR (95% CI)^2^
Children enrolled	79	41	76	37	
Median age at study visit in years (range)	2.7 (0.8–8.9)	2.8 (0.5–7.9)	5.4 (1.7–13.0)	6.3 (2.1–11.8)	
Number of male children	45 (57%)	22 (54%)	42 (55%)	19 (51%)	
**Nasopharyngeal carriage; n (%, 95% CI)**					
*Streptococcus pneumoniae*	47 (59, 48–70)	28 (68, 52–82)	44 (58, 46–69)	18 (49, 32–66)	1.45 (0.66–3.21)
*Haemophilus influenza*	47 (59, 48–70)	31 (76, 60–88)	32 (42, 31–54)	18 (49, 32–66)	0.81 (0.36–1.81)
*Moraxella catarrhalis*	38 (48, 37–60)	25 (61, 45–76)	22 (29, 19–40)	23 (62, 45–78)	0.25 (0.11–0.58)*
*Staphylococcus aureus*	6 (8, 3–16)	3 (7, 2–20)	11 (14, 7–24)	10 (27, 14–44)	0.46 (0.17–1.20)
**Antibiotics received <2 weeks before swab collection; n (%, 95% CI)**					
Macrolide	31 (39, 28–51)	0 (0, 0–9)	19 (25, 16–36)	0 (0, 0–9)	
Beta-lactam	15 (19, 11–29)	3 (7, 2–20)	12 (16, 8–26)	1 (3, 0–14)	

* P = 0.001; CI, confidence interval.

1 Swabs from 7 children who only ever had one swab collected (included in baseline) were excluded.

2 Multiple logistic regression compared carriage at the end of the study in Australian versus Alaskan children, adjusting for carriage at baseline.

### Association between frequency of azithromycin use and carriage

The number of children and time in the study were comparable when Australian children were grouped by azithromycin exposure (Table S1). However, there was a significant difference in the frequency of visits by group: Azi-None: median 3.5 (range 1–10); Azi-Infrequent: median 5 (range 2–14); Azi-Frequent: median 7 (range 1–15); nptrend P = 0.014. The median proportion of visits at which children had received azithromycin was 33% (range 12–50) in the Azi-Infrequent group and 75% (range 57–100) in the Azi-Frequent group. Differences in bacterial carriage among these groups were apparent at baseline. These differences were accentuated by the end of the study and were significant for all four bacteria (Table S1). A ‘cumulative dose-response’ relationship was observed: increasing azithromycin use coincided with decreasing carriage of *S. pneumoniae*, *H. influenzae* and *M. catarrhalis*, and increasing carriage of *S. aureus*. The same trends were apparent when data from all swabs were included (Figure S1, nptrend P<0.001 for all four bacteria).

Since azithromycin use coincided with carriage differences in the Australian children, carriage in the Alaskan children was compared with carriage in the Australian Azi-None group. Carriage of *S. pneumoniae* was higher in the Australian children, while *S. aureus* carriage was higher in Alaskan children. These differences were significant when all data were included; *S. pneumoniae* and *S. aureus* carriage were 79% (95% CI 71–87) and 2% (95% CI 0–6) respectively in the Australian Azi-None group compared to 60% (95% CI 52–67) and 15% (95% CI 9–21) respectively in Alaskan children.

### Antibiotic resistance

**Figure 2:**
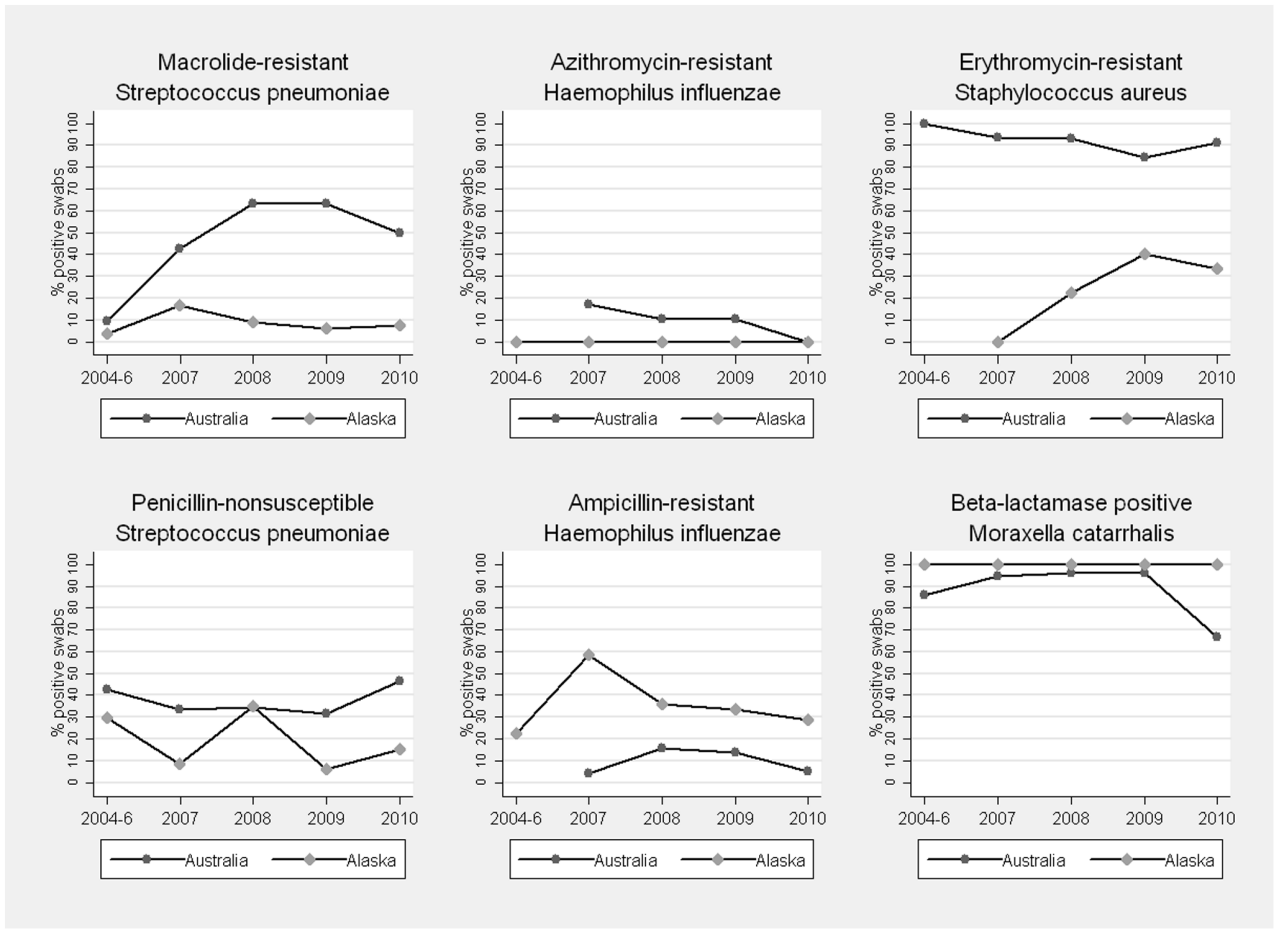
Pathogen resistance (proportion of carriers) by study year in Australian and Alaskan children.

Resistance of bacterial pathogens as children entered and left the study is shown in Table 3 and as a proportion of positive swabs by year in Figure 2. No penicillin resistant *S. pneumoniae* isolates (MIC>2 mg/L) were detected in either cohort. Macrolide resistance in *S. pneumoniae* and *S. aureus* carriers was significantly higher in Australian compared to Alaskan children. However, beta-lactam antibiotic resistance was higher in Alaskan *H. influenzae* carriers. Macrolide-resistant strains of *S. pneumoniae* and *S. aureus* were also more prevalent in Australian children when all swabs were included in analysis, isolated from 30% (95% CI 24–36) and 14% (95% CI 9–20) of swabs respectively, compared to 4.5% (95% CI 1–8) and 4% (95% CI 0–8) respectively in Alaskan children. *H. influenzae* and *M. catarrhalis* beta-lactam antibiotic resistance were both higher in Alaskan children as a proportion of all swabs: 19% (95% CI 13–26) had ampicillin-resistant *H. influenzae* and 65% (95% CI 56–74) had beta-lactamase positive *M. catarrhalis* strains compared to 4% (95% CI 2–6) and 29% (95% CI 23–35) respectively in Australian children.

**Table 3:** Antibiotic resistance (proportion of carriers) in respiratory bacteria from Australian and Alaskan children at baseline and end of study.

	First swab for each child		Last swab for each child	
	Australia	Alaska	Australia	Alaska
**Macrolide resistance; n (%, 95% CI)**				
MacR^1^ *S. pneumoniae*	17 (36, 23–51)	2 (7, 1–24)**	21 (48, 32–63)	2 (11, 1–35)**
AziIR^2^ *H. influenzae*	40 (85, 72–94)	not determined^3^	30^4^ (94, 79–99)	not determined^3^
AziR^2^ *H. influenzae*	7 (15, 6–28)	0 (0, 0–11) 3	1^4^ (3, 0–16)	0 (0, 0–19) 3
EryR^5^ *S. aureus*	5 (83, 36–100)	2 (67, 9–99)	10 (91, 59–100)	4 (40, 12–74)*
**Beta-lactam antibiotic resistance; n (%, 95% CI)**				
PenIR^6^ *S. pneumoniae*	22 (47, 32–62)	6 (21, 8–41)*	18 (41, 26–57)	4 (22, 6–48)
AmpR^7^ *H. influenzae*	7 (15, 6–28)	9 (29, 14–48)	3 (9, 2–25)	6 (33, 13–59)*
BLpos^8^ *H. influenzae*	6 (13, 5–26)	9 (29, 14–48)	3 (9, 2–25)	6 (33, 13–59)*
BLpos^8^ *M. catarrhalis*	35 (92, 79–98)	25 (100, 86–100)	22 (100, 85–100)	23 (100, 85–100)
MethR^9^ *S. aureus*	3 (50, 12–88)	0 (0, 0–70)	1 (9, 0–41)	2 (20, 3–56)

* P<0.05, ** P<0.01 for difference in resistance between Australian and Alaskan carriers; CI, confidence interval.

1 MacR, macrolide-resistant: azithromycin (Australia) and erythromycin (Alaska) minimum inhibitory concentration (MIC) >0.5 mg/L.

2 AziIR, azithromycin intermediate resistant: MIC >0.12–4 mg/L; AziR, azithromycin resistant: MIC >4 mg/L for Australian children.

3 All isolates from Alaskan children were susceptible on disk diffusion; MICs were not determined.

4 Isolates from one swab did not grow on sensitivity plates.

5 EryR, erythromycin resistant on disk diffusion.

6 PenIR, penicillin intermediate resistant: MIC >0.06–2 mg/L; no resistant (MIC >2 mg/L) isolates were detected in either cohort.

7 AmpR, ampicillin MIC >1 mg/L.

8 BLpos, beta-lactamase positive.

9 MethR, methicillin resistant on disk diffusion.

Resistance as a proportion of all isolates is reported in Table 4. All *H. influenzae* isolates from Alaskan children and 80% from Australian children were macrolide-susceptible by disk diffusion. Since most *H. influenzae* possess an intrinsic macrolide efflux mechanism [10] which confers intermediate resistance by EUCAST criteria, MICs were determined for all Australian isolates.

**Table 4:** Antibiotic resistance as percentage (95% CI) of total isolates in respiratory bacteria from Australian Indigenous and Alaska Native children.

	Australia	Alaska
***Streptococcus pneumoniae***	**285 isolates**	**96 isolates**
Macrolide resistant^1^	49 (43–55)	7 (2–12)
Penicillin intermediate resistant^2^	33 (28–39)	21 (13–29)
***Haemophilus influenzae***	**191 isolates**	**100 isolates**
Azithromycin intermediate resistant^3^	90 (86–95)	not determined
Azithromycin resistant	10 (5–14)^4^	0 (0–4)^5^
Ampicillin resistant^6^	11 (6–15)	32 (23–41)
Beta-lactamase positive	10 (6–14)	25 (17–33)
***Moraxella catarrhalis***	**146 isolates**	**100 isolates**
Beta-lactamase positive	88 (81–93)	100 (96–100)
***Staphylococcus aureus***	**70 isolates**	**23 isolates**
Erythromycin resistant^5^	91 (85–98)	26 (8–44)
Methicillin resistant^5^	17 (9–28)	13 (3–34)

CI, confidence interval.

1 Azithromycin or erythromycin MIC>0.5 mg/L; ^2^MIC>0.06–2 mg/L; ^3^MIC>0.12–4 mg/L;

4 MIC>4 mg/L; ^5^Resistant on disk diffusion; ^6^MIC>1 mg/L.

### Association between frequency of azithromycin use and resistance

Differences in bacterial resistance in the first and last swabs from Australian children grouped by azithromycin exposure are shown in Table S2. Macrolide resistance tended to be highest in the Azi-Frequent group for *S. pneumoniae*, *H. influenzae* and *S. aureus* carriers, but increased in *S. pneumoniae* in all three groups from the beginning to the end of the study. When all swabs were included, macrolide-resistant *S. pneumoniae* and *S. aureus* strains were found in 21% (95% CI 14–28) and 1% (95% CI 0–5) respectively from the Azi-None group, 28% (95% CI 17–38) and 14% (95% CI 6–22) from the Azi-Infrequent group, and 38% (95% CI 26–49) and 23% (95% CI 14–33) from the Azi-Frequent group (nptrend P = 0.002 and P<0.001 respectively). There were no trends for beta-lactam antibiotic resistance in any of the four pathogens. Trends by study year are shown in Figure S2.

### *S. pneumoniae* and *H. influenzae* serotypes and resistance patterns

**Figure 3:**
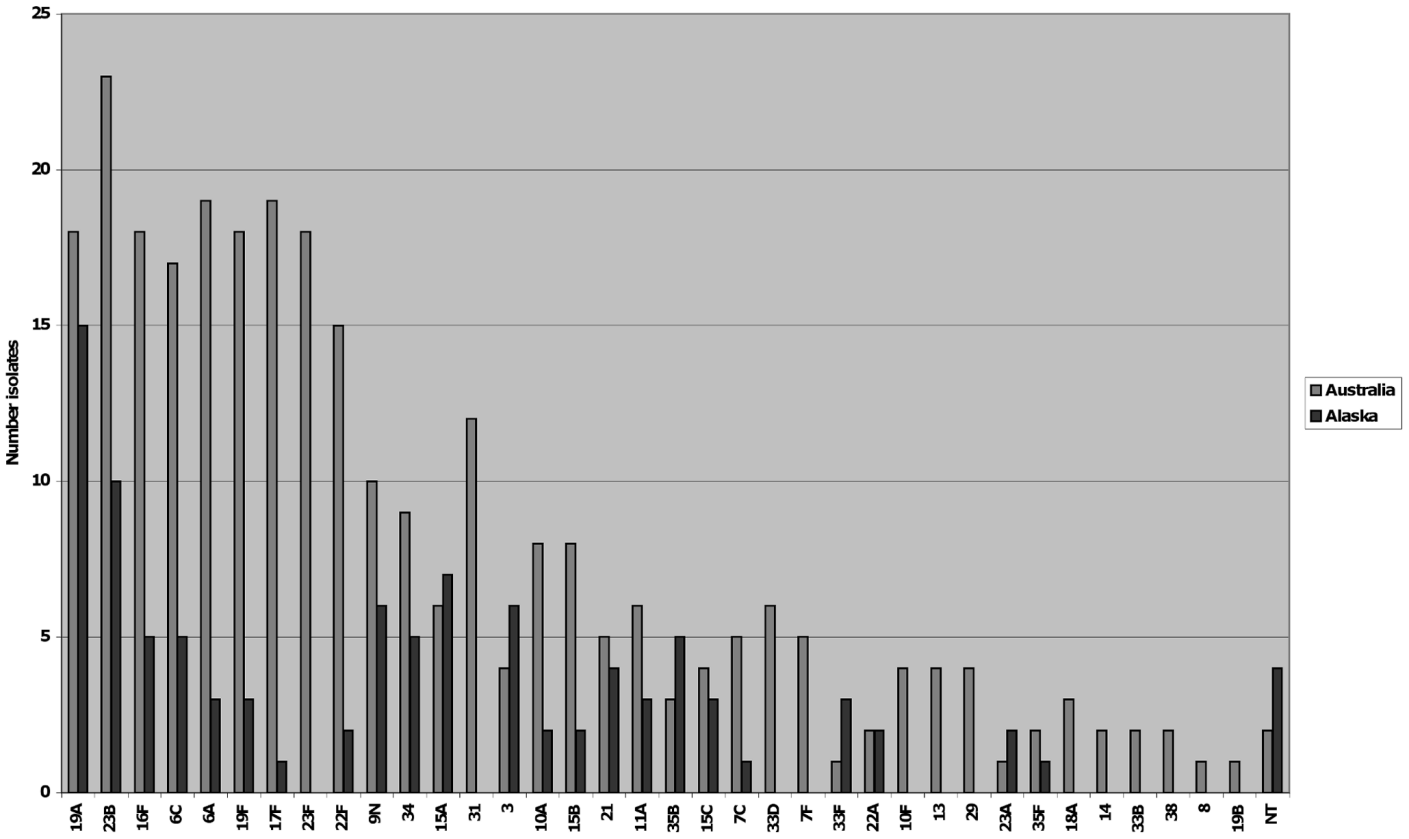
*Streptococcus pneumoniae* serotype hierarchy (ordered by combined totals) for 79 Australian and 41 Alaskan children. NT, nontypeable.

Of 285 *S. pneumoniae* isolates serotyped from the Australian children, PCV7 serotypes 19F and 23F were among the top 10 (Figure 3). The most prevalent serotype (8% of isolates) was 23B, followed by 6A, 19A and 6C (each 7%), and 16F, 17F, 19F and 23F (each 6.5%). Of 96 isolates serotyped from the Alaskan children the most prevalent serotype was 19A (15% of isolates) followed by 23B (9%) and 3, 15A and 34 (each 6.5%). The serotype hierarchy in Australian children appeared to coincide with azithromycin use; serotypes that were prevalent in the Azi-Frequent group also had the highest prevalence of azithromycin resistance. Commonly carried macrolide-resistant serotypes in the Australian group were 23B, 6A, 6C, 17F, 23F, 22F, 9N, 15A and 33D. In Alaskan children, azithromycin resistance was found in only 7 isolates (5 different serotypes). Intermediate penicillin resistance (MIC>0.06–2 mg/L) was most prevalent in serotypes 19A (100%), 19F, 23B (70% were both macrolide and intermediate penicillin resistant) and 16F in Australian children, and serotypes 19A (67%) and 35B in Alaskan children. Multiple strains were detected in 30 (12%) positive specimens (two had three serotypes) from Australian children and four (4%) from Alaskan children; this difference was likely due to more colonies being selected in Australia. Overall, PCV7 serotypes comprised 13% of Australian and 3% of Alaskan isolates.

Most *H. influenzae* isolates from both cohorts were nontypeable (NTHi) (Australia 89% of 191 isolates, Alaska 83% of 100 isolates). Capsular type b (Hib) was isolated from four children in both cohorts (2% and 4% of isolates respectively); three (75%) Hib isolates from Alaskan children were ampicillin resistant, but none from Australian children. Using EUCAST criteria, three isolates from Australian and eight from Alaskan children (three and seven NTHi respectively, no Hib) were beta-lactamase negative, but ampicillin resistant (BLNAR). However, only one BLNAR NTHi isolate from an Alaskan child had ampicillin MIC≥4 mg/L. Multiple strains (different serotype and/or antibiotype) were detected in 14% of Australian and 11% of Alaskan children.

### Associations between age and other bacteria and carriage

In bivariate analyses adjusted for repeated sampling of children, age was significantly associated with carriage. Older children were less likely to carry *H. influenzae* (Odds Ratio (OR) 0.91 per additional year of age, 95% CI 0.83–0.99), but more likely to carry *S. aureus* (OR 1.13 per additional year of age, 95% CI 1.01–1.25). These associations largely remained when study year was included in the analysis: OR 0.94 (95% CI 0.86–1.02) for *H. influenzae* and OR 1.11 (95% CI 0.99–1.25) for *S. aureus*.

In multivariate analyses adjusted for the presence of other respiratory bacteria and for repeated sampling of children, similar associations were seen in both cohorts for *S. pneumoniae* colonization, which was positively associated with *H. influenzae* (OR 3.18, 95% CI 2.07–4.88) and *M. catarrhalis* (OR 3.86, 95% CI 2.41–6.19) and negatively associated with *S. aureus* (OR 0.38, 95% CI 0.21–0.70). Similar associations were also seen for *H. influenzae* which was positively associated with the presence of *M. catarrhalis* (OR 3.27, 95% CI 2.21–4.81), and for *M. catarrhalis* which was negatively associated with *S. aureus* (OR 0.55, 95% CI 0.31–0.99).

## Discussion

This is the first longitudinal study reporting nasopharyngeal carriage in children with CSLD/bronchiectasis. While the two patient populations described are distinct and geographically dispersed, they share surprisingly similar environmental risk factors [1]. Carriage of respiratory pathogens in these two populations receiving different antibiotic treatment strategies showed similarities, but also important differences. *S. pneumoniae* carriage was similar overall and relatively stable in both Alaskan and Australian children during the study period. Carriage of *H. influenzae* declined in both cohorts over time, while *S. aureus* carriage increased. Carriage of *M. catarrhalis* in Alaskan children remained high throughout the study period, while carriage in Australian children started at the same level, but declined dramatically. Despite broadly similar carriage of three of the four main bacteria in the two populations, azithromycin use in the Australian cohort coincided with significant differences in the carriage of all four organisms, with an apparent ‘cumulative dose-response’ effect.

In the Australian Azi-None group, carriage of *S. pneumoniae* (79% of all swabs) was equivalent to that previously reported in this population [11]. Carriage of *S. pneumoniae* has been reported in several populations to peak at 2–3 years of age and decline thereafter [12], [13]. However, in Indigenous children and children from developing countries, carriage is higher than in developed countries and remains common up to 10-years of age [14], [15]. In the Azi-None group, carriage of *H. influenzae* was lower (60%) than the 85% reported in a younger cohort of Indigenous children [3], but similar to that reported for Aboriginal children 5–8 years of age [14]. The observed decline in carriage in both cohorts may therefore be age-related (as we found in bivariate analysis) as children grew older during the study. *M. catarrhalis* carriage (63%) in the Australian Azi-None group was lower than previously reported, even in older Indigenous children [14]. Nevertheless, *M. catarrhalis* carriage in this group and the Alaskan children was higher than generally reported in healthy children or children with upper respiratory tract infections [16].

*S. aureus* carriage has not been reported previously in this Australian population, and Alaskan reports are from a community with high rates of methicillin-resistant *S. aureus* infection [17]. In other populations, *S. aureus* carriage is high in infants <3-months of age, but declines rapidly as carriage of the other three bacteria increases, reaching a low point at 1–2 years [18], [19]. Carriage then increases again to reach its highest prevalence in children 6–11 years of age [12], [20]. If these trends apply to Australian and Alaskan children, then *S. aureus* carriage could be expected to be near its lowest point at the age when most of the children in this study were enrolled and highest at the age when their last swabs were collected. This would explain the observed increase in *S. aureus* carriage over the study period in both cohorts.

Consistent with other studies [3], [5], we found that azithromycin use coincided with significant reductions in carriage of *S. pneumoniae*, *H. influenzae* and *M. catarrhalis* in the Australian children. Our study also found an apparent ‘cumulative dose response’ effect on carriage and resistance. However, a potential confounding factor was attendance at clinic, as children who did not take azithromycin had fewer clinic attendances (fewer swabs collected). While we cannot exclude the possibility that they only attended clinic when they were sick and therefore more likely to be carrying bacteria, most swabs were collected at scheduled visits that were arranged independently of the child's health status. Factors other than azithromycin may also be involved in the decline of *M. catarrhalis* carriage in Australian children, since this decline was also seen in the Azi-None group. Conversely, while the increase in *S. aureus* carriage in Alaskan children is most likely age-related, the increase in Australian children may also be partly attributable to azithromycin since no such increase was seen in the Azi-None group (where *S. pneumoniae* carriage remained high).

Levels of macrolide resistance in bacteria colonizing the Australian children were not only higher than in the Alaskan children, but also higher than previously reported for most *S. pneumoniae* and *H. influenzae* paediatric respiratory tract isolates worldwide [10], [21], [22], and similar to the high levels of macrolide resistance in *S. aureus* often found in CF patients treated with azithromycin [23], [24]. Levels of beta-lactam antibiotic resistance in Alaskan children were similar to previous reports from North America [21], [22], and higher than *H. influenzae* and *M. catarrhalis* (but not *S. pneumoniae*) beta-lactam antibiotic resistance in Australian children. Beta-lactamase produced by one organism may protect co-colonizing bacteria from beta-lactam antibiotics [25], [26]. However, we found conflicting evidence using simple multivariate analyses (data not shown). The higher rates of intermediate resistance to penicillin in Australian *S. pneumoniae* isolates may be due to other factors, e.g. co-occurrence of macrolide and intermediate penicillin resistance in prevalent serotypes such as 23B.

As with other observational studies, it is possible that the associations observed may be confounded by factors not measured. Patients who took azithromycin consistently may differ from those who did not. Antibiotics taken before enrolment likely affected baseline carriage. Additionally, biases may have been introduced if the carriage status of children for whom we achieved good follow-up was systemically different to those for whom our follow-up was less successful. The different swabbing schedules (quarterly in Australia and annually in Alaska) may also have led to bias, e.g. if carriage varied seasonally and children were mostly seen at a particular time of year, or if resistant strains were persistently carried by the same child. However, children from both cohorts were seen in all months of the year, and a recent Kenyan study found the mean duration of *S. pneumoniae* carriage to be just over 30-days [27]. Thus swabbing every 3-months is unlikely to detect the same *S. pneumoniae* carriage episode, and this was confirmed by inspection of our serotype data by child visit. However, without further strain discrimination we cannot comment on carriage duration in other species. Finally, while changes in *H. influenzae* and *S. aureus* carriage over time appeared to be age-related, differences could also be explained by other temporal changes.

In conclusion, this study adds important new information on long-term carriage and antibiotic resistance trends in Indigenous children with CSLD/bronchiectasis from two environmentally diverse regions of the world. While similar, possibly age-related, changes in carriage were observed over time in both patient populations, azithromycin use was associated with a ‘cumulative dose-dependent’ pattern of carriage and resistance in the Australian children. Moreover, while two recent randomised controlled trials showed that adults with non-CF bronchiectasis had fewer respiratory exacerbations after receiving azithromycin continuously for 6–12 month periods [28], [29], this was accompanied by increased macrolide resistance amongst respiratory pathogens [29]. Such studies need to be repeated in children and, in light of these findings in adults and our own studies in children, special attention should be paid to monitoring antibiotic resistance as this may ultimately offset any clinical benefit derived from this important class of antibiotics.

## Supporting Information

Figure S1 **Pathogen carriage (proportion of swabs) by study year in Australian and Alaskan children.^1^** ^1^Australian children were grouped by proportion of study visits with azithromycin use <2-weeks before swab collection at: Azi-None  =  no study visits; Azi-Infreq(uent)  = 1–50% of study visits; Azi-Freq(uent)  = 51–100% of study visits.
(TIF)

Figure S2 **Pathogen resistance (proportion of carriers) by study year in Australian and Alaskan children.^1^** ^1^Australian children were grouped by proportion of study visits with azithromycin use <2-weeks before swab collection at: Azi-None  =  no study visits; Azi-Infreq(uent)  = 1–50% of study visits; Azi-Freq(uent)  = 51–100% of study visits.
(TIF)

Table S1 **Nasopharyngeal carriage of respiratory bacteria from Australian children, grouped by azithromycin exposure^1^, at baseline and end of study.** ^1^Australian children were grouped by proportion of study visits with azithromycin use <2-weeks before swab collection at: Azi-None  =  no study visits; Azi-Infreq(uent)  = 1–50% of study visits; Azi-Freq(uent)  = 51–100% of study visits.
(DOC)

Table S2 **Antibiotic resistance (proportion of carriers) in respiratory bacteria from Australian children, grouped by azithromycin exposure^1^, at baseline and end of study.** ^1^Australian children were grouped by proportion of study visits with azithromycin use <2-weeks before swab collection at: Azi-None  =  no study visits; Azi-Infreq(uent)  =  1–50% of study visits; Azi-Freq(uent)  = 51–100% of study visits.
(DOC)
