# Supplementary figures and images for: Longitudinal Nasopharyngeal Carriage and Antibiotic Resistance of Respiratory Bacteria in Indigenous Australian and Alaska Native Children with Bronchiectasis
Source: PLoS One. 2013 Aug 5;8(8):e70478. doi: 10.1371/journal.pone.0070478 (PMC3734249; doi:10.1371/journal.pone.0070478)

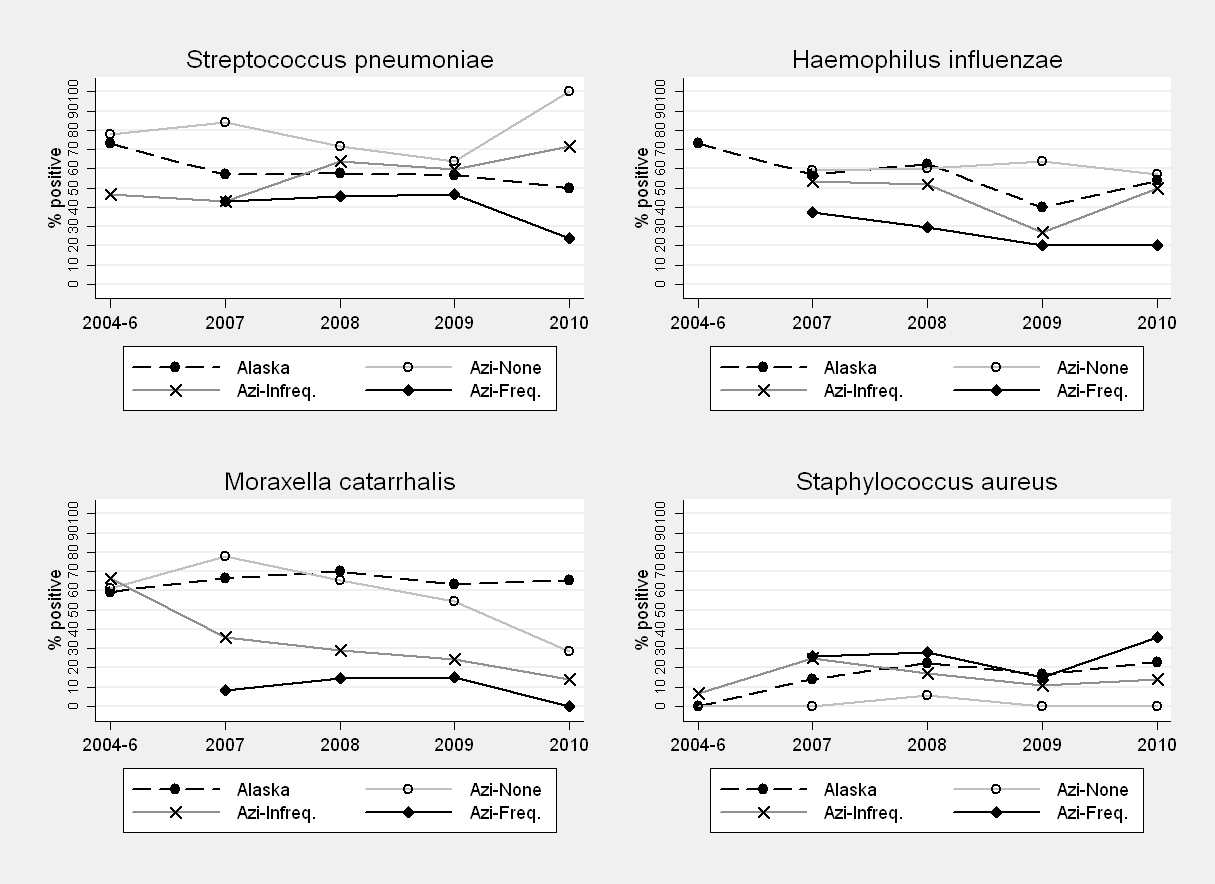

Supplement: Figure S1 — Pathogen carriage (proportion of swabs) by study year in Australian and Alaskan children.1 1Australian children were grouped by proportion of study visits with azithromycin use <2-weeks before swab collection at: Azi-None = no study visits; Azi-Infreq(uent) = 1–50% of study visits; Azi-Freq(uent) = 51–100% of study visits. (TIF) [file pone.0070478.s001.tif]

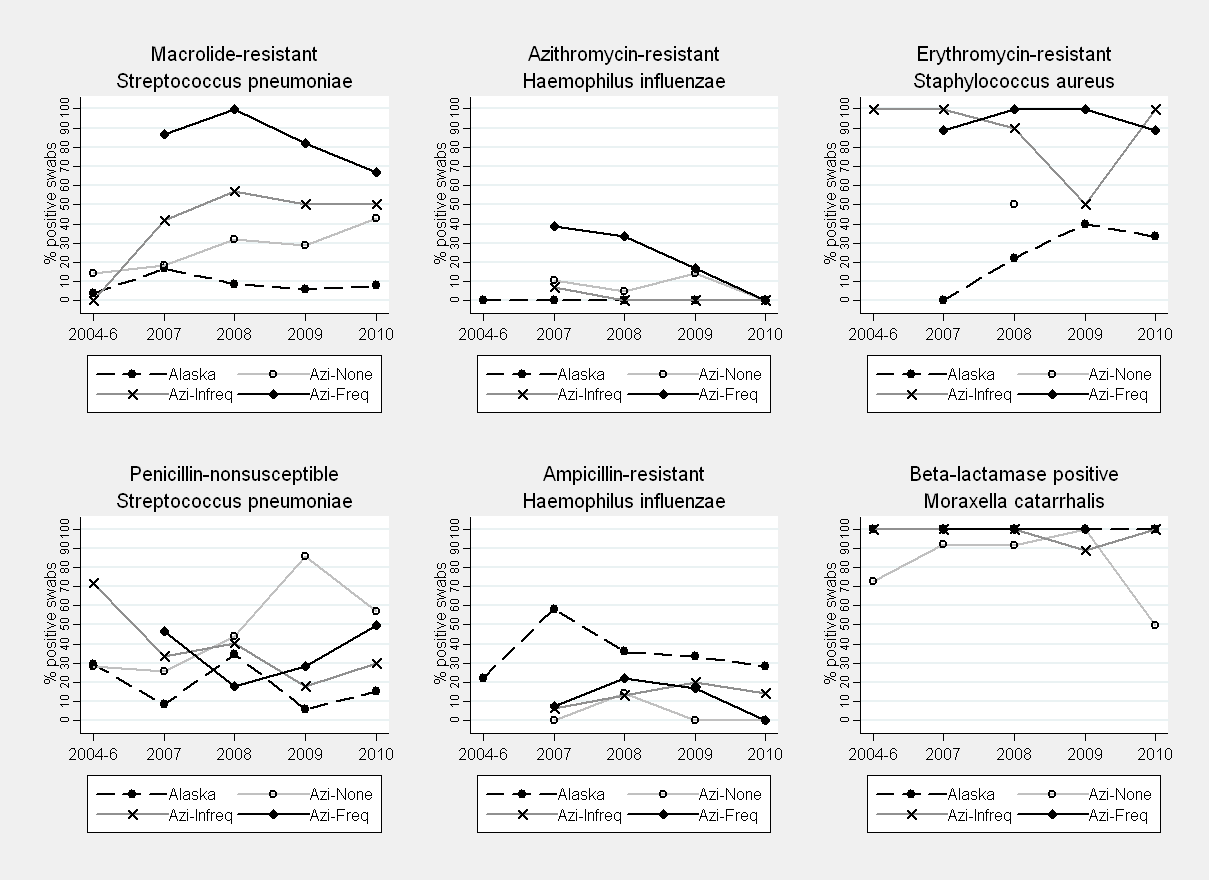

Supplement: Figure S2 — Pathogen resistance (proportion of carriers) by study year in Australian and Alaskan children.1 1Australian children were grouped by proportion of study visits with azithromycin use <2-weeks before swab collection at: Azi-None = no study visits; Azi-Infreq(uent) = 1–50% of study visits; Azi-Freq(uent) = 51–100% of study visits. (TIF) [file pone.0070478.s002.tif]
