# Supplementary material for: Longitudinal Nasopharyngeal Carriage and Antibiotic Resistance of Respiratory Bacteria in Indigenous Australian and Alaska Native Children with Bronchiectasis
Source: PLoS One. 2013 Aug 5;8(8):e70478. doi: 10.1371/journal.pone.0070478 (PMC3734249; doi:10.1371/journal.pone.0070478)
Supplement: Table S1 — Nasopharyngeal carriage of respiratory bacteria from Australian children, grouped by azithromycin exposure1, at baseline and end of study. 1Australian children were grouped by proportion of study visits with azithromycin use <2-weeks before swab collection at: Azi-None = no study visits; Azi-Infreq(uent) = 1–50% of study visits; Azi-Freq(uent) = 51–100% of study visits. (DOC) [file pone.0070478.s003.doc]

**Table S1. Nasopharyngeal carriage of respiratory bacteria from Australian children, grouped by azithromycin exposure^1^, at baseline and end of study.**

|  | **First swab for each child** | | | | **Last swab for each child^2^** | | | | |
| --- | --- | --- | --- | --- | --- | --- | --- | --- | --- |
|  | **Azi-None** | **Azi-Infrequent** | | **Azi-Frequent** | **Azi-None** | **Azi-Infrequent** | | | **Azi-Frequent** |
| Children enrolled | 26 | 26 | | 27 | 25 | 26 | | | 25 |
| Median age at study visit in years (range) | 3.3 (0.8-8.9) | 2.6 (1.0-8.5) | | 2.8 (1.1-8.6) | 6.2 (1.7-13.0) | 4.4 (2.6-12.9) | | | 4.6 (1.9-10.9) |
| Male sex; n (%) | 15 (58%) | 12 (46%) | | 18 (67%) | 14 (56%) | 12 (46%) | | | 16 (64%) |
| Median time in study in years (range) at last swab | | | | | 2.5 (0-5.8) | 2.1 (0.5-5.8) | | | 2.0 (0-3.7) |
| **Nasopharyngeal carriage; n (%, 95% CI)** | | | | | | | | | |
| *Streptococcus pneumoniae* | 21 (81, 61-93) | | 12 (46, 27-67) | 14 (52, 32-71)* | 21 (84, 64-95) | | 16 (62, 41-80) | | 7 (28, 12-49)*** |
| *Haemophilus influenzae* | 17 (65, 44-83) | | 18 (69, 48-86) | 12 (44, 25-65) | 16 (64, 43-82) | | 12 (46, 27-67) | | 4 (16, 5-36)*** |
| *Moraxella catarrhalis* | 17 (65, 44-83) | | 15 (58, 37-77) | 6 (22, 9-42)** | 13 (52, 31-72) | | 7 (27, 12-48) | | 2 (8, 1-26)*** |
| *Staphylococcus aureus* | 0 (0, 0-13) | | 3 (12, 2-30) | 3 (11, 2-29) | 2 (8, 1-26) | | 1 (4, 0-20) | | 8 (32, 15-54)* |
| **Antibiotics received <2 weeks before swab collection; n (%, 95% CI)** | | | | | | | | | |
| Macrolide | 0 (0, 0-13) | | 9 (35, 17-56) | 22 (81, 62-94) | 0 (0, 0-14) | 4 (15, 4-35) | | 15 (60, 39-79) | |
| Beta-lactam | 7 (27, 12-48) | | 7 (27, 12-48) | 1 (4, 0-19) | 6 (24, 6-42) | 2 (8, 1-25) | | 4 (16, 5-36) | |

* P<0.05, ** P<0.01, *** P≤0.001 for trend over 3 groups; CI, confidence interval.

^1^Australian children were grouped by proportion of study visits with azithromycin use <2-weeks before swab collection: Azi-None=no azithromycin at any study visit; Azi-Infrequent=azithromycin at 1-50% of study visits; Azi-Frequent=azithromycin at 51-100% of study visits.

^2^ Swabs from 3 children who only ever had one swab collected (included in baseline) were excluded.
