# Supplementary material for: Longitudinal Nasopharyngeal Carriage and Antibiotic Resistance of Respiratory Bacteria in Indigenous Australian and Alaska Native Children with Bronchiectasis
Source: PLoS One. 2013 Aug 5;8(8):e70478. doi: 10.1371/journal.pone.0070478 (PMC3734249; doi:10.1371/journal.pone.0070478)
Supplement: Table S2 — Antibiotic resistance (proportion of carriers) in respiratory bacteria from Australian children, grouped by azithromycin exposure1, at baseline and end of study. 1Australian children were grouped by proportion of study visits with azithromycin use <2-weeks before swab collection at: Azi-None = no study visits; Azi-Infreq(uent) = 1–50% of study visits; Azi-Freq(uent) = 51–100% of study visits. (DOC) [file pone.0070478.s004.doc]

**Table S2. Antibiotic resistance (proportion of carriers) in respiratory bacteria from Australian children, grouped by azithromycin exposure^1^, at baseline and end of study.**

|  | **First swab for each child** | | | **Last swab for each child** | | |
| --- | --- | --- | --- | --- | --- | --- |
|  | **Azi-None** | **Azi-Infrequent** | **Azi-Frequent** | **Azi-None** | **Azi-Infrequent** | **Azi-Frequent** |
| **Macrolide resistance; n (%, 95% CI)** | | | | | | |
| MacR^2^ *S. pneumoniae* | 4 (19, 5-42) | 2 (17, 2-48) | 11 (79, 49-95)* | 7 (33, 15-57) | 8 (50, 25-75) | 6 (86, 42-100)* |
| AziIR^3^ *H. influenzae* | 15 (88, 64-99) | 16^4^ (89, 65-99) | 9^5^ (75, 43-95) | 16 (100, 79-100) | 12 (100, 74-100) | 2^4^ (50, 7-93)* |
| AziR^3^ *H. influenzae* | 2 (12, 1-36) | 1^4^ (6, 0-27) | 4^5^ (33, 10-65)# | 0 (0, 0-21) | 0 (0, 0-26) | 1^4^ (25, 1-81)# |
| EryR^6^ *S. aureus* | none carried | 3 (100, 29-100) | 2 (67, 9-99)# | 1 (50, 1-99) | 1 (100, 3-100) | 8 (100, 63-100)# |
| **Beta-lactam antibiotic resistance; n (%, 95% CI)** | | | | | | |
| PenIR^7^ *S. pneumoniae* | 10 (48, 26-70) | 7 (58, 28-85) | 5 (36, 13-65) | 9 (43, 22-66) | 6 (38, 15-65) | 3 (43, 10-82) |
| AmpR^8^ *H. influenzae* | 1 (6, 0-29) | 3 (17, 4-41) | 3 (25, 5-57)# | 1 (6, 0-30) | 2 (17, 2-48) | 0 (0, 0-60)# |
| BLpos^9^ *H. influenzae* | 0 (0, 0-20) | 3 (17, 4-41) | 3 (25, 5-57)# | 1 (6, 0-30) | 2 (17, 2-48) | 0 (0, 0-60)# |
| BLpos^9^ *M. catarrhalis* | 14 (82, 57-96) | 15 (100, 78-100) | 6 (100, 54-100) | 13 (100, 75-100) | 7 (100, 59-100) | 2 (100, 16-100) |
| MethR^10^ *S. aureus* | none carried | 2 (67) [9-99) | 1 (33, 1-91)# | 0 (0, 0-84) | 0 (0, 0-98) | 1 (13, 0-53)# |

* P<0.05 for trend over 3 groups; # statistical tests not performed due to small numbers; CI, confidence interval.

^1^Australian children were grouped by proportion of study visits with azithromycin use <2-weeks before swab collection: Azi-None=no azithromycin at any study visit; Azi-Infrequent=azithromycin at 1-50% of study visits; Azi-Frequent=azithromycin at 51-100% of study visits.

^2^MacR, macrolide-resistant: azithromycin (Australia) and erythromycin (Alaska) minimum inhibitory concentration (MIC) >0.5 mg/L.

^3^AziIR, azithromycin intermediate resistant: MIC >0.12-4 mg/L; AziR, azithromycin resistant: MIC >4 mg/L.

^4^Isolates from one swab did not grow on sensitivity plates.

^5^One swab had AziIR and AziR strains of *H. influenzae*.

^6^EryR, erythromycin resistant on disk diffusion.

^7^PenIR, penicillin intermediate resistant: MIC >0.06-2 mg/L; no resistant (MIC >2 mg/L) isolates were detected in any group.

^8^AmpR, ampicillin MIC >1 mg/L.

^9^BLpos, beta-lactamase positive.

^10^MethR, methicillin resistant on disk diffusion.
